# Changes of monocyte human leukocyte antigen-DR expression as a reliable predictor of mortality in severe sepsis

**DOI:** 10.1186/cc10457

**Published:** 2011-09-20

**Authors:** Jian-Feng Wu, Jie Ma, Juan Chen, Bin Ou-Yang, Min-Ying Chen, Li-Fen Li, Yong-Jun Liu, Ai-Hua Lin, Xiang-Dong Guan

**Affiliations:** 1 Department of Surgical Intensive Care Unit, The First Affiliated Hospital of Sun Yat-sen University, 58 Zhongshan Er Road, Guangzhou 510080, PR China; 2 Department of Medical Statistics and Epidemiology, School of Public Health, Sun Yat-sen University, 74 Zhongshan Er Road, Guangzhou 510080, PR China

## Abstract

**Introduction:**

Many studies have shown that monocyte human leukocyte antigen-DR (mHLA-DR) expression may be a good predictor for mortality in severe septic patients. On the contrary, other studies found mHLA-DR was not a useful prognostic marker in severe sepsis. Few studies have taken changes of mHLA-DR during treatment into consideration. The objective of this study was to estimate the prognostic value of changes of mHLA-DR to predict mortality in severe sepsis.

**Methods:**

In this prospective observational study, mHLA-DR was measured by flow cytometry in peripheral blood from 79 adult patients with severe sepsis. mHLA-DR levels were determined on day 0, 3, 7 after admission to the surgical intensive care unit (SICU) with a diagnosis of severe sepsis. ΔmHLA-DR_3 _and ΔmHLA-DR_7 _were defined as the changes in mHLA-DR value on day 3 and day 7 compared to that on day 0. Data were compared between 28-day survivors and non-survivors. Receiver operating characteristic (ROC) curves were plotted to measure the performance and discriminating threshold of ΔmHLA-DR_3_, ΔmHLA-DR_7_, ΔmHLA-DR_7-3_, mHLA-DR_0_, mHLA-DR_3 _and mHLA-DR_7 _in predicting mortality of severe sepsis.

**Results:**

ROC curve analysis showed that ΔmHLA-DR_3 _and ΔmHLA-DR_7 _were reliable indicators of mortality in severe sepsis. A ΔmHLA-DR_3 _value of 4.8% allowed discrimination between survivors and non-survivors with a sensitivity of 89.0% and a specificity of 93.7%; similarly, ΔmHLA-DR_7 _value of 9% allowed discrimination between survivors and non-survivors with a sensitivity of 85.7% and a specificity of 90.0%. Patients with ΔmHLA-DR_3 _≤4.8% had higher mortality than those with ΔmHLA-DR_3 _> 4.8% (71.4% *vs*. 2.0%, OR 125.00, 95% CI 13.93 to 1121.67); patients with ΔmHLA-DR_7 _≤9% had higher mortality than those with ΔmHLA-DR_7 _> 9% (52.9% *vs*. 2.0%, OR 54.00, 95% CI 5.99 to 486.08). The mean change of mHLA-DR significantly increased in the survivor group with the passage of time; from day 0 to day 3 and day 7, changes were 6.45 and 16.90 (*P *< 0.05), respectively.

**Conclusions:**

The change of mHLA-DR over time may be a reliable predictor for mortality in patients with severe sepsis.

## Introduction

Severe sepsis is an important cause of admission to intensive care units (ICUs) throughout the world and is characterized by high mortality in adults [1-4]. But the pathogenesis of sepsis is still not clear. Previous reports suggest that the depression of the immune system may contribute to the severity of sepsis. Although the mechanistic and molecular bases for ICU-acquired immunosuppression are not exhaustively established, several features of the condition, including enhanced leukocyte apoptosis, lymphocyte anergy, and deactivated monocyte functions, have already been described [5-7]. mHLA-DR has been suggested to be a reliable marker for estimating immunosuppression. The level of mHLA-DR was significantly decreased during severe sepsis [8], although little is known about the underlying mechanism. The reduced expression was associated with high mortality and had a predictive value for the prognosis of patients with sepsis [9-12] or the risk of secondary infection [13-16]. However, the association between low mHLA-DR and mortality in severe sepsis has been challenged [17]. These differences in findings may be partially explained by the fact that immune function is dynamically changing during the clinical course of severe sepsis. In this study, we monitored the expression of mHLA-DR during 1 week to evaluate the predictive power of serial determinations of mHLA-DR as a marker of mortality in severe sepsis. Our hypothesis was that ΔmHLA-DR would be more accurate than mHLA-DR in predicting 28-day mortality in severe sepsis.

## Material and methods

### Patients

This prospective single-center study was conducted in a surgical ICU (SICU) with 12 beds of a tertiary, teaching hospital between June 2008 and August 2010. A total of 107 patients were enrolled. Written informed consent was obtained from the patients or, for patients unable to provide consent, from their closest relatives. The study protocol was approved by the ethics committee of the hospital.

### Inclusion criteria

Inclusion criteria were (a) age of between 18 and 85 years and (b) admission to the SICU with a diagnosis of severe sepsis according to criteria of the American College of Chest Physicians/Society of Critical Care Medicine [18]. Exclusion criteria were (a) being pregnant or lactating; (b) receiving immunosuppressive therapy such as cyclosporine, azothioprine, or cancer-related chemotherapy; (c) expected survival of fewer than 28 days because of incorrectable medical conditions, such as poorly controlled neoplasm or other end-stage disease; (d) history of bone marrow, lung, liver, kidney, pancreas, or small bowel transplantation; and (e) acute pancreatitis with no established source of infection.

### Study design

Patients were screened on the admission day, and clinical and biological variables were collected. These included demographic characteristics (age and gender), microbiological findings (primary infection source and the identified microorganisms), and comorbidities (chronic obstructive pulmonary disease, chronic heart failure, malignant diseases, and diabetes). The primary outcome variable was mortality at day 28 (death or survival). The following clinical parameters were recorded: the initial severity as assessed by the Acute Physiology and Chronic Health Evaluation II (APACHE II) [19] and the Sequential Organ Failure Assessment (SOFA) (score range of 0 to 24) [20] on days 0 (on the day of ICU admission), 3, and 7. Blood samples for analysis of mHLA-DR were collected on days 0, 3, and 7, respectively. ΔmHLA-DR_3 _and ΔmHLA-DR_7 _were defined as the value change in mHLA-DR on days 3 and 7 compared with that on day 0 (mHLA-DR_0_), and ΔmHLA-DR_7-3 _was defined as the value change in mHLA-DR on day 7 compared with that on day 3. That is, ΔmHLA-DR_3 _= mHLA-DR_3 _- mHLA-DR_0_; ΔmHLA-DR_7 _= mHLA-DR_7 _- mHLA-DR_0_; and ΔmHLA-DR_7-3 _= mHLA-DR_7 _- mHLA-DR_3_.

### mHLA-DR measurement by flow cytometry

Expression of cell surface HLA-DR on monocytes was measured by flow cytometry (EPICSXL; Beckman Coulter, Inc., Fullerton, CA, USA). Staining and cell acquisition for flow cytometry were performed within 1 hour after blood sampling. Monoclonal antibodies were used as follows: CD14-PE (20 μL, clone M5E2; BD Biosciences, San Jose, CA, USA) and HLA-DR-FITC (20 μL, clone G46-6; BD Biosciences) per 100 μL of whole blood. Negative controls were mouse monoclonal antibodies IgG2a-PE (clone G155-178) and IgG2a-FITC (clone G155-178), which were isotype-matched in accordance with the recommendations of the manufacturer. Monocytes were characterized on the basis of their CD14 expression. At least 1,500 monocytes were analyzed from each sample. Results are expressed as percentages of HLA-DR-positive monocytes out of the total monocyte population.

### Statistical analysis

Quantitative variables with normal distribution were expressed as mean ± standard deviation, and quantitative variables with non-normal distribution were expressed as median and interquartile range (IQR). To estimate mean changes in mHLA-DR for the survivor and non-survivor groups, as well as corresponding between-group differences of changes in mHLA-DR, linear mixed models with random patient effects were employed. This analysis took into account the clustering of repeated measures in patients and the baseline (day 0) mHLA-DR and included all available cases. The survival estimate was based on the Kaplan-Meier method, and comparisons of survival distributions were based on the log-rank test. Receiver operating characteristic (ROC) curves were produced for ΔmHLA-DR_3_, ΔmHLA-DR_7_, ΔmHLA-DR_7-3_, mHLA-DR_0_, mHLA-DR_3_, and mHLA-DR_7 _to determine the discriminating threshold of each parameter. The optimal cutoff points were determined by maximizing the sum of sensitivity and specificity. The areas under the ROC curves (± standard error) were calculated for each parameter, and the comparison between AUCs was conducted on 'fully paired' case samples in accordance with the non-parametric approach reported by DeLong and colleagues [21]. The Pearson chi-square test or Fisher exact test, as appropriate, was used for categorical data. Univariate and multivariate logistic regressions were used to identify the variables associated with the risk of death assessed by odds ratios (ORs) and their 95% confidence intervals (CIs). The variables with a *P *value of not more than 0.10 in univariate analysis were entered in the multivariate adjusted model. The predictors included demographic and clinical parameters. Multivariate regression analysis was then performed for the independent variables by 'enter' method. Given that different ΔmHLA-DRs were not independent variables, they were entered into the model separately. A *P *value of less than 0.05 was considered statistically significant. The analyses were performed with SPSS software (version 15.0; SPSS Inc., Chicago, IL, USA).

## Results

### Baseline and clinical characteristics of the patients

**Figure 1:**
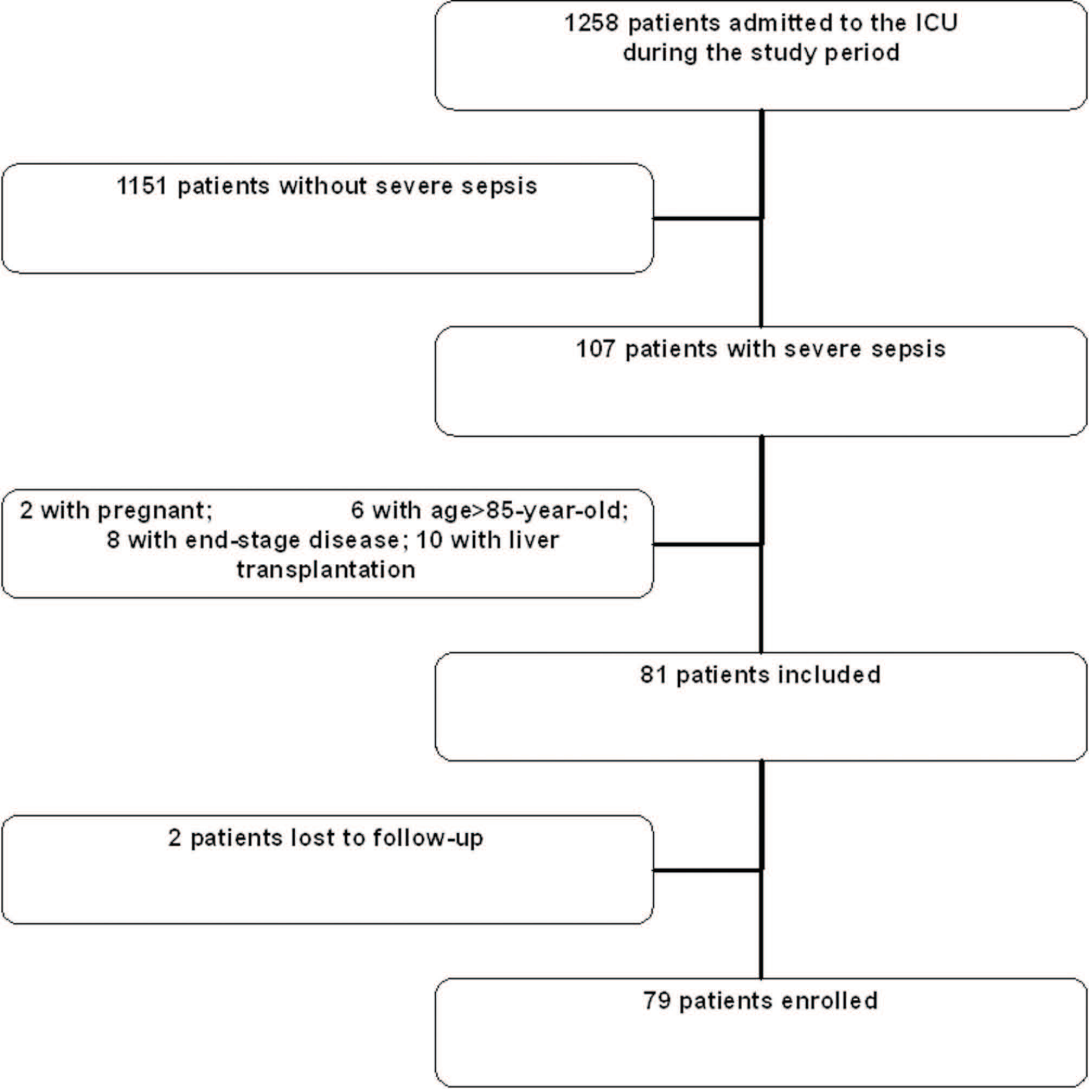
**Trial profile**. ICU, intensive care unit.

During the study period, 107 patients with severe sepsis were admitted to the SICU. Six patients did not meet the entry criterion regarding age limitation, 20 patients met at least one exclusion criterion, and 2 patients were lost to follow-up (Figure 1). Overall, 79 patients were enrolled in the study (63 males and 16 females). The ages ranged from 23 to 75 years (average age of 61 ± 13.6 years), APACHE II score within 24 hours of SICU admission was a median of 19 (IQR 8), and the median SOFA score was 8 (IQR 5). The most common sites of infection were lungs and abdomen, accounting for 36 patients (45.6%) and 42 patients (53.2%), respectively. After admission, 7 patients died within 3 days and 13 other patients died within 7 days, leaving 79, 72, and 66 surviving patients on days 0, 3, and 7, respectively; overall 28-day mortality rate was 29.1% (23/79).

### Comparison of mHLA-DR expression changes between survivors and non-survivors

mHLA-DR was significantly increased in the survivor group with the passage of time. The mean changes from day 0 to day 3 and day 7 were 6.45 (*P *= 0.002) and 16.90 (*P *< 0.001), respectively. It was 10.45 (*P *< 0.001) from day 3 to 7. However, the non-survivor group presented no significant changes in mHLA-DR (*P *> 0.05). On day 3, the difference of change from day 0 to 3 in mHLA-DR between survivors and non-survivors was 8.14 and was of borderline significance (*P *= 0.053). On day 7, the corresponding differences were 12.35 (*P *= 0.038) from day 0 to 7 and 4.21 (*P *= 0.423) from day 3 to 7. These findings suggested that the survivors had a sustained increase in mHLA-DR over time and presented a significantly increasing tendency compared with the non-survivors (Table 1).

**Table 1:** Linear mixed model on the mean (95% confidence interval) changes in mHLA-DR at every measure point in the survivor and non-survivor groups

	Day 0		Day 3		Day 7				
	**Number**	**mHLA-DR**	**Number**	**mHLA-DR**	**Number**	**mHLA-DR**	**ΔmHLA-DR_3_**	**ΔmHLA-DR_7_**	**ΔmHLA-DR_7-3_**

Survivor	56	62.97 (56.38~69.55)	56	69.42 (62.92~75.92)	56	79.87 (73.96~85.78)	6.45^a^ (2.50~10.40)	16.90^b^ (11.85~21.95)	10.45^c^ (6.19~14.71)
Non-survivor	23	56.77 (46.49~67.05)	16	55.08 (44.26~65.90)	10	61.32 (49.79~72.84)	-1.69^d^ (-8.95~5.57)	4.55^e^ (-5.96~15.06)	6.24^f^ (-3.28~15.76)
Between- groups difference							8.14^g^ (-0.12~16.40)	12.35^h^ (0.70~24.00)	4.21^i^ (-6.21~14.64)

ΔmHLA-DR_3 _and ΔmHLA-DR_7 _were defined as the value change in mHLA-DR on days 3 and 7 compared with that on day 0, and ΔmHLA-DR_7-3 _was defined as the value change in mHLA-DR on day 7 compared with that on day 3. In the comparison of mean mHLA-DR differences in the same group between every measurement point, *P *values were ^a^0.002, ^b ^< 0.001, ^c ^< 0.001, ^d^0.644, ^e^0.392, and ^f^0.195. In the comparison of ΔmHLA-DR differences between two groups within the same measurement point, *P *values were ^g^0.053, ^h^0.038, and ^i^0.423. CI, confidence interval; mHLA-DR, monocyte human leukocyte antigen-DR.

### Prognostic value of the changes of mHLA-DR expression

**Figure 2:**
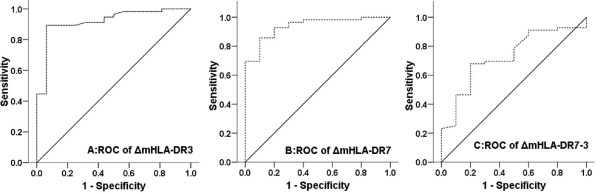
**Receiver operating characteristic (ROC) of ΔmHLA-DR_3_, ΔmHLA-DR_7_, and ΔmHLA-DR_7-3_**. ROC analysis depicted the discriminating value of **(a) **ΔmHLA-DR_3_, **(b) **ΔmHLA-DR_7_, and **(c) **ΔmHLA-DR_7-3 _for 28-day mortality in severe sepsis. ΔmHLA-DR_3 _and ΔmHLA-DR_7 _were defined as the value change in mHLA-DR on days 3 and 7 compared with that on day 0, and ΔmHLA-DR_7-3 _was defined as the value change in mHLA-DR on day 7 compared with that on day 3. mHLA-DR, monocyte human leukocyte antigen-DR.

**Figure 3:**
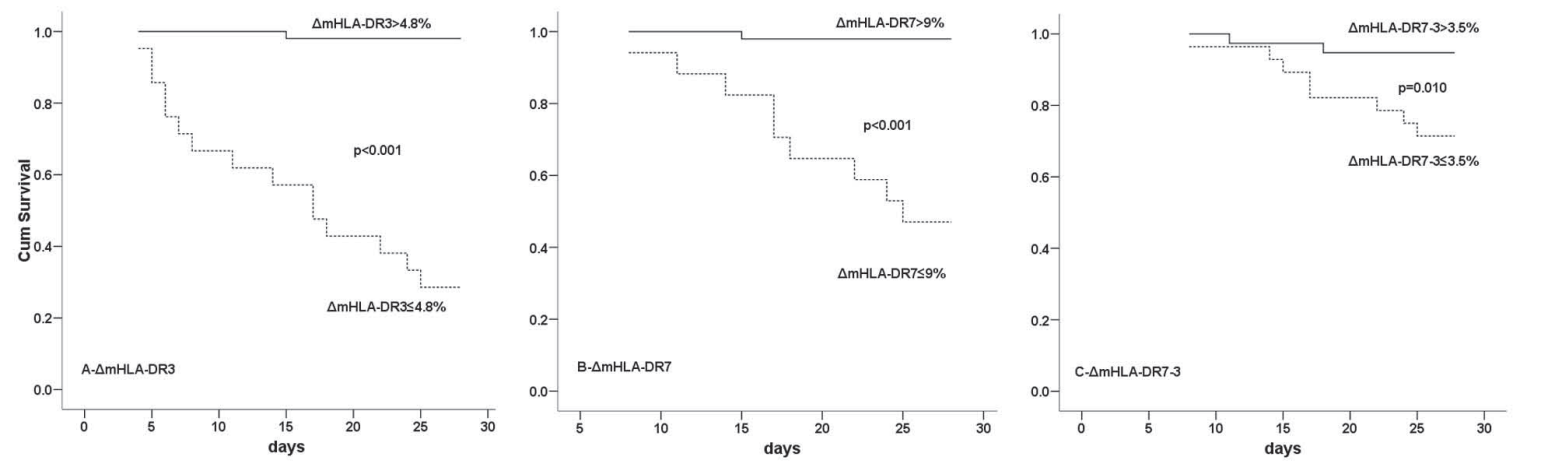
**Survival curves of ΔmHLA-DR**. Comparison of 28-day mortality by means of Kaplan-Meier survival curves for **(a) **patients with ΔmHLA-DR_3 _of greater than 4.8% (continuous line) and ΔmHLA-DR_3 _of not more than 4.8% (dashed line), **(b) **patients with ΔmHLA-DR_7 _of greater than 9% (continuous line) and ΔmHLA-DR_7 _of not more than 9% (dashed line), and **(c) **patients with ΔmHLA-DR_7-3 _of greater than 3.5% (continuous line) and ΔmHLA-DR_7-3 _of not more than 3.5% (dashed line). ΔmHLA-DR_3 _and ΔmHLA-DR_7 _were defined as the value change in mHLA-DR on days 3 and 7 compared with that on day 0, and ΔmHLA-DR_7-3 _was defined as the value change in mHLA-DR on day 7 compared with that on day 3. mHLA-DR, monocyte human leukocyte antigen-DR.

The areas under the ROC curve (AUCs) of ΔmHLA-DR_3_, ΔmHLA-DR_7_, and ΔmHLA-DR_7-3 _for 28-day mortality were 0.919 (*P *< 0.001), 0.938 (*P *< 0.001), and 0.729 (*P *= 0.022), respectively; ΔmHLA-DR_3_, ΔmHLA-DR_7_, and ΔmHLA-DR_7-3 _all had high specificity and sensitivity for prediction of mortality. The AUCs of mHLA-DR_0_, mHLA-DR_3_, and mHLA-DR_7 _were 0.570 (*P *> 0.05), 0.629 (*P *> 0.05), and 0.598 (*P *> 0.05), respectively. mHLA-DR_0_, mHLA-DR_3_, and mHLA-DR_7 _all had low specificity for prediction of mortality but had high sensitivity (Table 2 and Figures 2 and 3). The difference in AUC between ΔmHLA-DR_3 _and mHLA-DR_3 _is 0.289 ± 0.077 (95% CI 0.139 to 0.439, *P *< 0.001), the difference in AUC between ΔmHLA-DR_7 _and mHLA-DR_7 _is 0.350 ± 0.091 (95% CI 0.172 to 0.528, *P *< 0.001), and the difference between ΔmHLA-DR_7-3 _and mHLA-DR_7 _is 0.140 ± 0.110 (95% CI -0.076 to 0.356, *P *= 0.204).

**Table 2:** Predictive value for 28-day mortality of ΔmHLA-DR and mHLA-DR

Number of patients	Variables	Cutoff value	Sensitivity	Specificity	Positive predictive value	Negative predictive value	AUC	95% CI	*P *value
72	ΔmHLA-DR_3_	4.8%	89.0%	93.7%	70.9%	98.0%	0.919 ± 0.032	0.83-0.97	< 0.001
66	ΔmHLA-DR_7_	9.0%	85.7%	90.0%	60.5%	97.2%	0.938 ± 0.030	0.851-0.982	< 0.001
66	ΔmHLA-DR_7-3_	3.5%	66.1%	80.0%	37.1%	92.9%	0.729 ± 0.079	0.573-0.884	0.022
79	mHLA-DR_0_	35.0%	89.1%	43.5%	62.1%	79.3%	0.570 ± 0.070	0.453-0.682	0.319
72	mHLA-DR_3_	39.5%	91.1%	37.5%	54.6%	83.6%	0.629 ± 0.075	0.508-0.740	0.116
66	mHLA-DR_7_	47.0%	94.6%	30.0%	49.8%	88.3%	0.598 ± 0.094	0.460-0.708	0.376

ΔmHLA-DR_3 _and ΔmHLA-DR_7 _were defined as the value change in mHLA-DR on days 3 and 7 compared with that on day 0, and ΔmHLA-DR_7-3 _was defined as the value change in mHLA-DR on day 7 compared with that on day 3; mHLA-DR_0_, mHLA-DR_3_, and mHLA-DR_7 _were defined as the value of mHLA-DR on days 0, 3, and 7. AUC, area under the curve; CI, confidence interval; mHLA-DR, monocyte human leukocyte antigen-DR.

There was a significant difference in mortality between patients with ΔmHLA-DR_3 _of not more than 4.8% (15/21) and those with ΔmHLA-DR_3 _of greater than 4.8% (1/51) (71.4% versus 2.0%, OR 125.00, 95% CI 13.93 to 1,121.67). Similarly, patients with ΔmHLA-DR_7 _of not more than 9% had a higher mortality rate (9/17) than those with ΔmHLA-DR_7 _of greater than 9% (1/49) (52.9% versus 2.0%, OR 54.00, 95% CI 5.99 to 486.08), and patients with ΔmHLA-DR_7-3 _of not more than 3.5% had a higher mortality rate (8/28) than those with ΔmHLA-DR_7-3 _of greater than 3.5% (2/38) (28.6% versus 5.3%, OR 7.20, 95% CI 1.39 to 37.23).

Gender and all ΔmHLA-DRs were entered in the multivariate regression analysis because their *P *values were lower than 0.1 in univariate analysis. Given that different ΔmHLA-DRs were correlated with each other (a circumstance that would lead to collinearity if they were in the same model), different ΔmHLA-DRs were entered into the multivariate logistic regression model separately. After being adjusted for gender, the results indicated that ΔmHLA-DR_3 _(*P *< 0.001), ΔmHLA-DR_7 _(*P *< 0.001), and ΔmHLA-DR_7-3 _(*P *= 0.022) were associated with a higher mortality (Table 3).

**Table 3:** Univariate and multivariate logistic regression analysis used to differentiate survivors and non-survivors

		Univariate (*n *= 66)			Multivariate (*n *= 66)		
		OR	95% CI	*P *value	OR	95% CI	*P *value
Gender	Male	1.00	-	-			
	Female	5.22	1.25-21.82	0.023	8.19^a^	0.75-89.05	0.084
					4.56^b^	0.65-31.81	0.126
					5.47^c^	1.15-25.99	0.033
Age, years	≤62	1.00	-	-			
	> 62	2.51	0.59-10.69	0.214			
APACHE II score	≤18	1.00	-	-			
	> 18	2.53	0.63-9.89	0.192			
SOFA score	≤7	1.00	-	-			
	> 7	2.02	0.48-8.63	0.341			
ΔmHLA-DR_3_	> 4.8	1.00	-	-		-	
	≤4.8	75.00	8.04-699.44	< 0.001	94.71^a^	7.64-1174.27	< 0.001
ΔmHLA-DR_7_	> 9	1.00	-	-			
	≤9	54.00	5.99-486.08	< 0.001	51.04^b^	5.35-486.94	< 0.001
ΔmHLA-DR_7-3_	> 3.5	1.00					
	≤3.5	7.20	1.39-37.23	0.019	7.46^c^	1.34-41.39	0.022

^a^ΔmHLA-DR_3_, ^b^ΔmHLA-DR_7_, and ^c^ΔmHLA-DR_7-3 _were entered into multivariate logistic regression model separately with adjustment of gender. ΔmHLA-DR_3 _and ΔmHLA-DR_7 _were defined as the value change in mHLA-DR on days 3 and 7 compared with that on day 0, and ΔmHLA-DR_7-3 _was defined as the value change in mHLA-DR on day 7 compared with that on day 3. APACHE II, Acute Physiology and Chronic Health Evaluation II; CI, confidence interval; OR, odds ratio; SOFA, Sequential Organ Failure Assessment.

### Comparison of 28-day mortality in patients grouped by mHLA-DR expression with 30% as a cutoff value

The 28-day mortality of patients with mHLA-DR expression of not more than 30% and those greater than 30% on days 0, 3, and 7 was compared, respectively. There was no significant difference in 28-day mortality between patients with mHLA-DR expression of not more than 30% and those greater than 30% on day 0 (50% (5/10) versus 26.1% (18/69), *P *= 0.120), day 3 (37.5% (3/8) versus 20.3% (13/64), *P *= 0.364), and day 7 (25.0% (1/4) versus 14.5% (9/62), *P *= 0.490), although non-survivors tended to exhibit lower mHLA-DR expression than survivors.

## Discussion

Sepsis is one of the 10 leading causes of death in critically ill patients in the US. It is the third leading cause of death among patients in non-coronary ICUs [22,23]. Severe sepsis develops each year in more than 750,000 people, 215,000 of whom die of the disease [3], and is considered a disorder partly due to immunosuppression [5]. The diagnosis of immunosuppression depends on paraclinical parameters because of the absence of specific clinical symptoms. Among these, monocyte expression of human leukocyte antigen type DR (HLA-DR) has been shown to be useful for monitoring immunoparalysis and accepted as a reliable marker for evaluation of immune function [8,15,17]. Also, downregulation of mHLA-DR is generally accepted as a reliable marker for an immune dysfunction in patients with sepsis [24].

Volk and colleagues [12] were the first to describe immunoparalysis indicated by mHLA-DR expression in patients with sepsis. Abundant research has demonstrated the reduction of mHLA-DR expression in patients with sepsis [8,9,13]. A recent study indicated that patients in severe trauma present with a transient immunosuppression with decreased mHLA-DR expression. The lack of mHLA-DR recovery between days 3 and 4 and days 1 and 2 is associated with sepsis [25]. Furthermore, the prognostic value of low HLA-DR expression on monocytes has been elucidated, and the severity of the sepsis and mortality have been correlated with low HLA-DR expression [8,10,15,26,27]. In recent years, it has been shown that patients with sepsis-induced immunosuppression were at a higher risk to develop secondary infections [13]. In a prospective single-center observational trial, Landelle *et al*. found that persistently low mHLA-DR expression is independently associated with the development of nosocomial infections [14,28]. Monocyte HLA-DR expression has also been successfully applied to monitor immunomodulatory therapies, including medications such as granulocyte/macrophage colony-stimulating factor (GM-CSF) [29,30], filgrastim [31], thymosin alpha 1 [32], and interferon-gamma [33], as well as extracorporeal immune interventions such as immunoadsorption treatment and continuous hemodiafiltration [34,35].

In contrast, other research suggested that mHLA-DR was not a useful prognostic marker for outcome. A single-center study showed no significant difference in mortality between patients with low HLA-DR expression and those with normal HLA-DR expression [17]. That study indicated that mHLA-DR did not give satisfactory discriminatory power to assist in an outcome prediction. Another study reported that the low HLA-DR expression was not an independent outcome predictor, because the correlation between outcome and early HLA-DR expression disappeared after adjustment of the severity of illness by Sequential Organ Failure Assessment score or Simplified Acute Physiology Score II [13]. The contradictory results prompt clinicians to seek a more representative index for the connection between immune status and outcomes.

Although a number of recent studies have adopted static HLA-DR expression as a predictive marker, few of them have addressed the changes of HLA-DR expression during the disease progress. To address the fact that the immune function is dynamically influenced by severe sepsis and to compensate for the drawbacks of previous studies, we measured the expression of mHLA-DR consecutively to find out whether its change over time could predict mortality.

Our results indicated that mHLA-DR was significantly increased in the survivor group with the passage of time, but not in the non-survivor group. The findings were similar to those of Monneret and colleagues [27]. In a study in septic shock, they found that mHLA-DR expressions were not significantly different between survivors and non-survivors at days 1 and 2. However, at days 3 and 4, the mHLA-DR expression had increased in survivors, but not in non-survivors.

We found that the AUCs of mHLA-DR_3 _and mHLA-DR_7 _for 28-day mortality in patients with severe sepsis were 0.629 and 0.598, respectively, with low specificity despite the relatively high sensitivity. In contrast, ΔmHLA-DR was a good predictor for the outcome of severe sepsis. Among patients with severe sepsis, those with increased ΔmHLA-DR expression higher than threshold had markedly lower mortality. ROC curve analysis showed that ΔmHLA-DR_3 _and ΔmHLA-DR_7 _were reliable indicators of mortality in severe sepsis with high sensitivity and specificity. Multivariate logistic regression analysis showed that ΔmHLA-DR_3_, ΔmHLA-DR_7_, and ΔmHLA-DR_7-3 _were all associated with a higher mortality. However, the wide range of CIs implied poor precision because of the relatively small size of the cohort. It was also found that ΔmHLA-DR_7-3 _was not as good as ΔmHLA-DR_7 _and ΔmHLA-DR_3 _either in predicting mortality or in representing the difference in change of mHLA-DR between survivors and non-survivors. A possible explanation is that ΔmHLA-DR_3 _and ΔmHLA-DR_7 _are calculated from a baseline on day 0 but that ΔmHLA-DR_7-3 _is calculated from a baseline on day 3, meaning that ΔmHLA-DR_7-3 _is affected by more confounding factors such as the treatment in the ICU, the deterioration or improvement of disease, and other conditions that have impacts on immune status. Overall, our study suggests that elevated ΔmHLA-DR expression (especially ΔmHLA-DR_3 _and ΔmHLA-DR_7_) may be seen as a marker for a gradually recovering immune function during the course of severe sepsis or a positive response to treatment and may indicate a better outcome.

A threshold of 30% is retained to predict mortality in published research that showed that non-survivors had an expression of mHLA-DR of lower than 30% [9,36]. However, in this study, there was no significant difference in 28-day mortality between patients with mHLA-DR expression of not more than 30% and those greater than 30% on days 0, 3, and 7, although non-survivors tended to exhibit lower mHLA-DR expression than survivors. At the same time, the present study found that mean expression of mHLA-DR in non-survivors was about 60%. This finding may be explained by the following facts: (a) our study included only surgical patients, who may have a relatively minor degree of immunosuppression compared with other critical patients with severe underlying diseases, and (b) to ensure that severe sepsis was the most likely cause for patients' immunosuppression in our study, we had excluded severely immnunosuppressed patients caused by other factors, including post-transplantation status and immunosuppressive therapy. It is assumed that different patient populations and selection criteria as well as the heterogeneity of septic host [8] may contribute to the varied findings in different studies. Given the mixed results in different studies, a fixed static value of HLA-DR may not be appropriate to be applied in all patient populations and the dynamic change of HLA-DR seems more reasonable as an index for mortality.

The present study has certain limitations that need to be taken into account. This is a single-center study with a relatively small cohort, and the findings need to be confirmed by a multicenter study. In our study, mHLA-DR was expressed as percentages of HLA-DR-positive monocytes in the total monocyte population, and the measurement was reported to be reproducible with coefficients of variation from precision studies less than 5% [37]. In spite of this, the value of delta HLA-DR should be separately interpreted because we cannot exclude the variability of measurements by flow cytometry. With the development of techniques, quantitative flow cytometry offers a better means of standardization within and between flow cytometers [38]. With this method, results become comparable among different laboratories.

Given these limitations, our objective is not to establish a golden standard about delta mHLA-DR for predicting severe sepsis mortality considering the small cohort and heterogeneity in severe sepsis but to remind clinicians that the dynamic change of mHLA-DR may be a better parameter than static values in judging prognosis and evaluating efficacy of immunomodulatory therapies. Actually, similar indices such as delta central venous pressure, delta stroke volume, and lactate clearance are being widely used in the ICU.

## Conclusions

Our findings demonstrate that, in severe sepsis, monitoring the changes of mHLA-DR over time might be beneficial to predict mortality in comparison with a single measurement of mHLA-DR. This may help to identify patients at increased risk of death.

## Key messages

• Whether mHLA-DR expression is a good predictor for mortality in patients with severe sepsis is controversial.

• In this prospective observational study, we monitored the changes of mHLA-DR expression in patients with severe sepsis.

• Our findings demonstrate that, in severe sepsis, monitoring the changes of mHLA-DR may be a better way to predict mortality than taking a single measurement of mHLA-DR.

## Abbreviations

APACHE II: Acute Physiology and Chronic Health Evaluation II; AUC: area under curve; CI: confidence interval; HLA-DR: human leukocyte antigen-DR; ICU: intensive care unit; IQR: interquartile range; mHLA-DR: monocyte human leukocyte antigen-DR; OR: odds ratio; ROC: receiver operating characteristic; SICU: surgical intensive care unit; SOFA: Sequential Organ Failure Assessment.

## Competing interests

The authors declare that they have no competing interests.

## Authors' contributions

J-FW and JM designed and performed the research and wrote the manuscript. X-DG designed the research, provided the supportive work and supervision, and wrote the manuscript. JC, BO-Y, M-YC, L-FL, and Y-JL performed the research. A-HL analyzed the data. All authors read and approved the final manuscript.
